# Metabolomics targets tissue-specific responses in alleviating the negative effects of salinity in tef (*Eragrostis tef*) during germination

**DOI:** 10.1007/s00425-023-04224-x

**Published:** 2023-08-19

**Authors:** Bahareh Sadat Haddadi, Rui Fang, Aiswarya Girija, Divya Kattupalli, Emma Widdowson, Manfred Beckmann, Rattan Yadav, Luis A. J. Mur

**Affiliations:** 1 Department of Life Sciences (DLS), Aberystwyth University, Wales, SY23 3DA UK; 2 Institute of Biological Rural Environmental Sciences (IBERS), Aberystwyth University, Wales, SY23 3EE UK

**Keywords:** *Eragrostis tef*, Flavonoids, Germination, Metabolomics, Root colour, Salinity

## Abstract

**Main conclusion:**

Salinity induced metabolite responses resulted in differential accumulation of flavonoids and antioxidant metabolites in shoots and roots suggesting improved antioxidant capacity in providing salt-adaptive phenotype of tef seedling.

**Abstract:**

Tef [(*Eragrostis tef*) (Zucc.) Trotter] is an important ‘cash crop’ of Ethiopia grown mainly for human food, and development of elite tef cultivars with better performance is vital to Ethiopian farmers and breeders. Soil salinity is one of the key constraints that affects tef yield in the Ethiopian lowlands and Rift valley where cultivation of tef is limited. Being a minor crop, the responses of tef towards salinity is unknown. Salinity involves physiological and metabolite reprogramming that can have major impact on germination and seedling establishment. Here we evaluate the in vitro effect of NaCl on tef germination and associate this with metabolomic approaches to suggest salt tolerance mechanisms. In this study, 19 tef varieties were screened for NaCl tolerance and were investigated using untargeted metabolomics. Screened tef varieties showed differential germination rates with NaCl treatment varying from < 20 to 100%. Viable seedlings exposed to NaCl exhibited purple-red pigment accumulation in the roots except for Beten and Tullu nasy varieties. Metabolite comparisons between shoots and roots showed significant differences and, in particular, roots of salt tolerant tef varieties accumulated flavonoid derivatives as well as sugars and cell wall associated metabolites. These metabolic changes were correlated with patterns of antioxidant capacities and total flavonoid content in shoots and roots and suggested a mitigating response by tef to salinity. Our study highlights the role of flavonoid accumulation following salt stress on tef seedlings and further these findings could be used as targets for selective tef breeding.

**Supplementary Information:**

The online version contains supplementary material available at 10.1007/s00425-023-04224-x.

## Introduction

Tef [(*Eragrostis tef*) (Zucc.) Trotter] is an orphan crop native to Ethiopia and has been cultivated in Ethiopia for more than 10,000 years (Assefa et al. 2015; Zhu 2018). Tef is a staple food crop that feeds for over 60 million people in the Horn of Africa and is cultivated annually on three million hectares of land which is about 28% of the total area covered by other cereals in Ethiopia (Lee 2018). After coffee, tef is the second most ‘cash crop’ and important export crop for Ethiopian farmers aiding its socio-economic development (Bachewe et al. 2019). In recent years, tef is gaining global attention as a ‘super-grain’ from nutritionists and consumers due to its health and nutritional benefits. Compared to other major cereals such as wheat, rice, barley, oats, maize, tef grains are high in amino acid, protein, fiber, vitamin, and mineral content (Zhu 2018; Girija et al. 2022b). Tef grains are also gluten free with low glycemic index which makes it an alternative cereal for people suffering from gluten intolerance and Type 2 diabetes (Ligaba-Osena et al. 2020, 2022). As a result, apart from Ethiopia, tef is cultivated as a food and forage crop in USA, Netherlands, South Africa, Australia, Indian, Kenya and Israel (Tadele and Hibistu 2021).

As a C_4_ crop tef is adapted to grow under adverse environmental conditions such as imposed by drought, salinity, and waterlogging and at elevations ranging from 1800 and 2200 m above sea level (Ereful et al. 2022; Girija et al. 2022a). Research into tef is limited (Numan et al. 2021), which is compromising it achieving its potential. For example, despite its large area of cultivation, tef yields in Ethiopia remain far below as compared to wheat and maize (Cochrane and Bekele 2018). Ethiopian institutes hold the largest tef collection with about more than 5000 diverse tef accessions collected from different agro-ecological zones. This could provide the resources for the derivation of elite tef genotypes (Girma et al. 2014; Fikre et al. 2018). The key environmental constraints that affect tef productivity are lodging, drought and salinity and high diversity between tef cultivars such as seed color, plant height, leaf traits and the relatively long life cycle which makes the screening of stress tolerance traits within tef cultivars difficult (Woldeyohannes et al. 2022). Thus, there is an urgent need to characterize the tef genetic resources for key traits as a prerequisite for their use in breeding programs.

Salinity is one of the major abiotic stresses affecting plant growth and crop productivity (Yang and Guo 2018). Seed germination is the first phase of a plant life cycle negatively affected by salinity (Bewley and Black 2013; Nawaz et al. 2022). During seed germination, excessive salinity brings about cytotoxicity through water stress and oxidative damage caused by excess sodium (Na^+^) and chloride (Cl^−^) ions, which ultimately leads to the inhibition and impairing of crop growth and development (El-Hendawy et al. 2019). This imbalance in ion homeostasis prevents cell division and elongation by restricting water absorption and inducing ion toxicity (Liu et al. 2022). Moreover, it reduces the activities of some key enzymes that can ultimately diminish the seed reserves to impact on germination and seedling establishment (Parihar et al. 2015). To counter this salinity triggers, several adaptive responses occur such as antioxidant defenses and wider gene-metabolite reprogramming to influence plants at molecular, metabolic and physiological levels (Liu et al. 2015).

Globally, about 831 million hectares of total land area is affected by soli salinization. In Ethiopia this is a particular problem in the lowland areas for example the large Rift and Awash Valleys (Asfaw and Danno 2011) and tef cultivation is limited in these regions due to the lack of salt tolerant varieties. Therefore, the derivation of salt tolerant elite tef varieties is an important aim for Ethiopian agriculture. However, this aim can be facilitated by a better understanding of stress adaptive traits and mechanisms in tef. Given this, we describe the metabolite responses of tef to salt during germination and identify tef lines with better germination tolerance under saline conditions. We target alterations in the metabolite pathways that could contribute towards salt resilience to tef during germination. Selecting genotypes which exhibit these changes could be important in the selection of breeding targets for developing tef varieties that can germinate and grow in saline enriched soil.

## Materials and methods

### Germination and salt treatment

In the initial experiment, 19 different tef varieties were used to screen for germination under salt stress (Table S1). Tef seeds were sterilized with 70% ethanol for 2 min, followed by a 10 min rinse in 5% sodium hypochlorite and then washed 5 times with distilled water. After sterilization, seeds of each genotype were germinated in 12 cm Petri dishes containing filter papers soaked with 7 mL of distilled water (control) or 100, 250 and 500 mM NaCl. Each Petri dish contained about 150 seeds and each seed was counted as a replicate. Seeds were germinated in a growth chamber under 25 °C/21 °C day/night 12 h cycle for 7 days. Germination was scored when the radicle approached 2 mm length and data were collected daily, and imaging of plates were done every day. At the end of the germination test, the mean final percentage of germination (GP) was calculated by the formula below:

$${\text{GP }} = {\text{ number of seeds germinated}}/{\text{total number of seeds tested }} \times { 1}00$$

After the initial screening, the 19 genotypes were classified as either salt sensitive or tolerant based on germination rates. No germination was observed at 250 and 500 mM NaCl. Germination of 19 genotypes was performed again under control (water) and 100 mM NaCl solution, and based on germination percentage these genotypes were selected as being representative of sensitive or tolerant genotypes. After scoring, the plant material was used for metabolite analysis.

### Metabolite analysis

Seedlings were harvested on the 7th day after germination and used for metabolite extraction. Each replicate represented a pool of fifteen seedlings with shoot and roots collected separately. The shoot and root samples (~ 7 mg ± 1 mg) of each tef accession (*n* = 3) were placed into 2 mL microcentrifuge tubes, each containing a single 5 mm diameter stainless-steel ball (acetone cleaned). Samples were immediately flash frozen in liquid N_2_ and homogenized using a ball mill, to which 250 µL of ice-cold extraction buffer (chloroform/methanol/water, 1:2.5:1, by vol.) was added followed by incubation at 4 °C for 15 min. The samples were further centrifuged at 21,000×*g* for 5 min at 4 °C to separate the supernatant. Metabolite fingerprinting was performed for 100 µL of the extracted samples by flow infusion electrospray ionization high-resolution mass spectrometry (FIE-HRMS) using a Q Exactive Plus Hybrid Quadrupole Orbitrap Mass Analyser with an Acella ultra high-performance liquid chromatography (UHPLC) system (Thermo Fisher Scientific, Bremen, Germany). The sample was injected into the capillary column in a randomized order and the *m/z* (mass-ion) features were generated in both positive and negative ionization mode. (The derived results are presented in Supplementary Tables S2 to S5).

### Data analysis

Individual *m/z* values were log_10_ transformed and used for multivariate analysis using R based platform MetaboAnalyst (http://www.metaboanalyst.ca, accessed August 2022). The significance of the cross validated *P* values, based on the one-way analysis of variance (ANOVA) was set to *P* < 0.05. The multiple comparison and post hoc test using Fisher’s Least Significant Difference (Fisher’s LSD) were performed. The functional level and pathway enrichment assessment were performed using the Functional analysis module of MetaboAnalyst 5.0. Metabolite identification was based on the MS peaks to pathway *mummichog* algorithm (tolerance = 5 ppm, reference library: *Oryza sativa*).

### Determination of antioxidant activity and total flavonoid content (TFC)

The antioxidant activity and flavonoid content in shoots and roots of tef seedlings was determined by 2,2-diphenyl-1-picrylhydrazyl (DPPH) free radical scavenging and TFC assays, respectively. In the DPPH free radical scavenging assay, shoots and roots were separated from tef seedlings germinated under control (water) and saline (NaCl) conditions and was extracted with 80% methanol. Ascorbic acid was used as standard and 300 µL of the extract was used for DPPH assay (Brand-Williams et al. 1995) and absorbance was measured at 515 nm using the Hidex Sense Plate Reader (Lab Logic, Sheffield, UK). For TFC aluminum chloride colorimetric assay was performed with quercetin (1 mg mL^−1^) (Golkar and Taghizadeh 2018) as standard and absorbance was measured at 510 nm using the Hidex Sense Plate Reader.

## Results

### Effect of salinity on germination and seedling growth

**Fig. 1:**
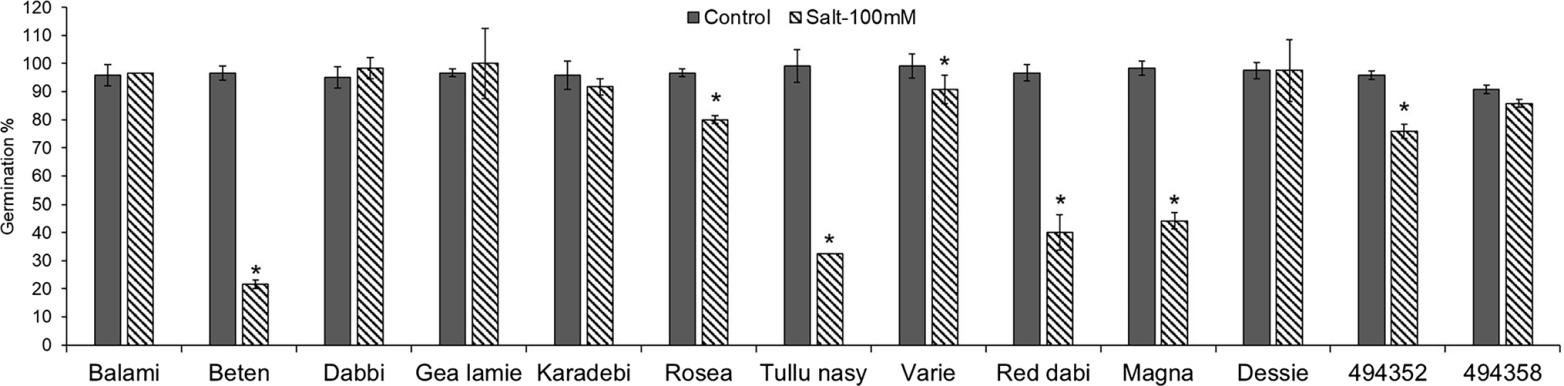
Effect of salinity stress on germination percentage in 13 tef genotypes under control (water) and salinity (100 mM NaCl). The graph represents the average germination percentage of tef on the 7th day after sowing. Data collected from 40 individual seeds, pooled as one biological replicate (*n* = 40, biological replicate 1). Data presented as average ± SD with three biological replicates. Statistical significance calculated by t test. *, *P* ≤ 0.05

**Fig. 2:**
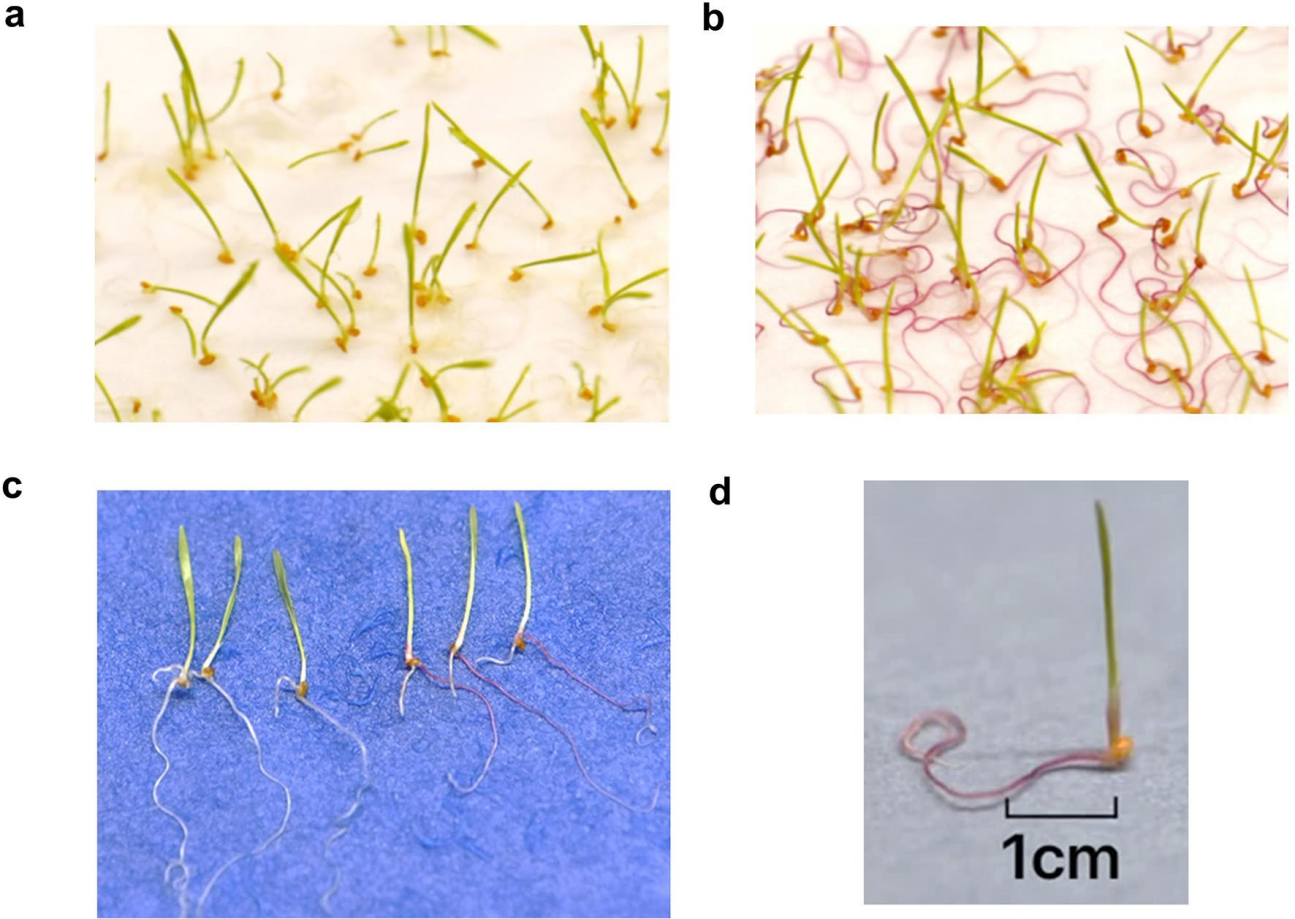
Tef seedlings germinated under control and saline conditions. **a** Tef seedlings germinated in water. **b** Tef seedlings germinated in 100 mM NaCl. **c** Comparison of three tef seedlings germinated each in control (from the left) and saline (100 mM) (from the right) conditions. **d** Tef seedling germinated in 100 mM NaCl showing purple-red pigment accumulation in root

An initial salt screening was performed to assess the germination of 19 different tef varieties under salt (NaCl) stress. Seeds were germinated in Petri dishes under three NaCl concentrations (100, 250 and 500 mM) and water as control. Germination was monitored for seven days, and germination was only observed in the control and 100 mM NaCl. A second germination assay was therefore performed with water and 100 mM salt. Based on the germination percentage, the tef genotypes were classified into tolerant (> 75%), susceptible (> 20–50%) and very susceptible (< 20%) (Fig. 1). Of the accessions screened, 6 genotypes failed to germinate in saline conditions, i.e., Addissie, Alba, Enatite, Gommadie, Manyi, 3910, and were classified as very susceptible (Table 1). These were omitted from further analysis. Compared to controls, Beten, Tullu nasy, Red dabi and Magna (20–50%) showed poor germination with salt (and were designated as susceptible) while all other genotypes showed germination frequencies > 75–90% (designated as tolerant). We observed a purple-red pigment accumulation in the roots of most of the tolerant tef genotypes germinated under saline conditions (Fig. 2, Table 2). The exceptions were Tullu nasy and Beten which were white in both control and salt treatments and Balami that showed root pigmentation under both treatments. To investigate the metabolite changes in shoots and roots, a tissue-specific metabolite analysis was carried out for the viable 13 genotypes.

**Table 1:** Classification of 19 tef genotypes as tolerant, susceptible, and very susceptible based on their germination percentage in 100 mM NaCl compared to the control (water)

Tolerant > 75%	Susceptible > 20–50%	Very susceptible < 20%
Balami Dabbi Gea lamie Karadebi Rosea Variegata Dessie 494352 494358	Beten Tullu nasy Red dabi Magna	Alba Addissie Manyi Enatite Gommadie 3910

**Table 2:** Root colour of Tef seedlings germinated in control (water) and saline (100 mM NaCl)

Genotypes	Control-water	100 mM NaCl
Balami	Purple-red	Purple-red
Beten	White	White
Dabbi	White	Purple-red
Gea-lamie	White	Purple-red
Karadebi	White	Purple-red
Rosea	White	Purple-red
Tullu nasy	White	White
Variegata	White	Purple-red
Red dabi	White	Purple-red
Magna	White	Purple-red
Dessie	White	Purple-red
494352	White	Purple-red
494358	White	Purple-red

### Untargeted metabolomics of tef seedings under salinity

Shoot and roots were separated from the tef seedlings, and an independent tissue-specific metabolite extraction was performed. A high-throughput untargeted metabolite profiling was carried out to understand the key metabolite alterations using FIE-HRMS.

### The shoot metabolome

**Fig. 3:**
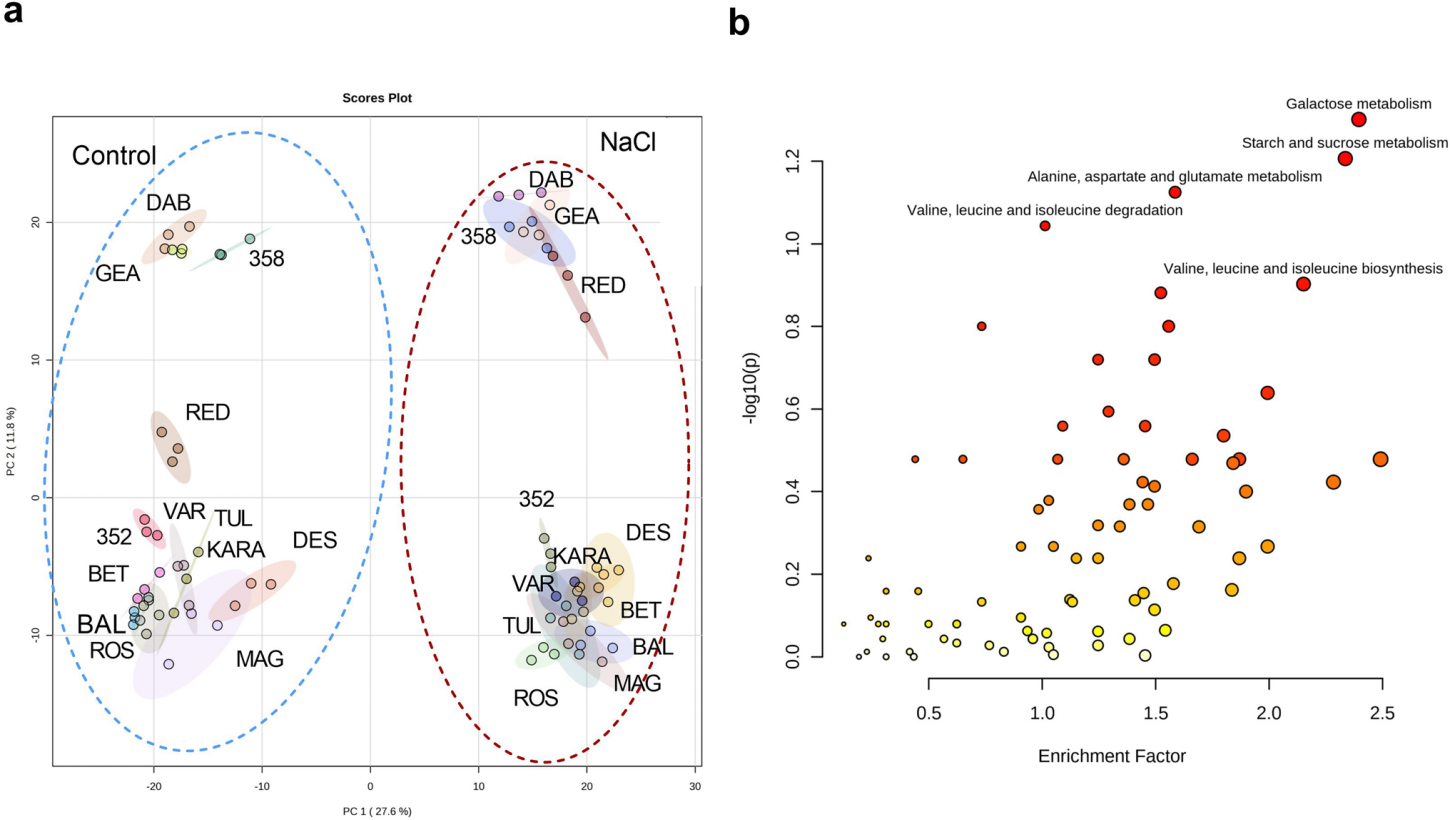
**a** Principal component analysis results for shoot metabolome of thirteen tef genotypes. Genotype abbreviations: 352 = 494352; 358 = 494358; BAL = Balami; BET = Beten; DAB = Dabbi; GEA = Gea-lamie; KARA = Karadebi; MAG = Magna; RED = Red dabi; ROS = Rosea; TUL = Tullu nasy; VAR = Variegata; Group labels (CS = control shoot, SS = salt shoot). The blue open circle indicates control samples, and the red open circle indicates salt (100 mM) treated samples. **b** Pathway enrichment of significant metabolite features found in shoot metabolome of tef seedlings

**Fig. 4:**
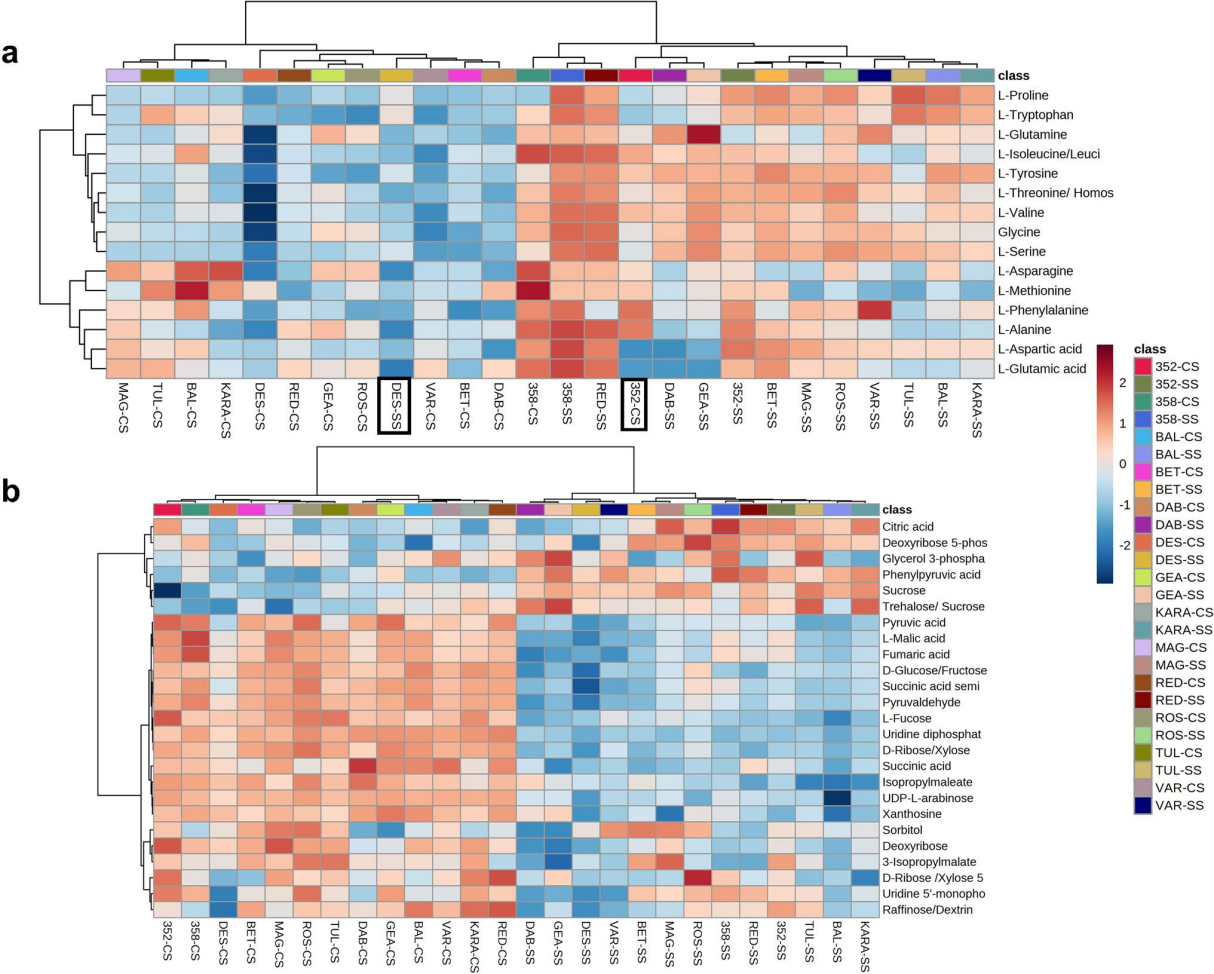
Heat maps of metabolite classes present in the shoot of tef seedlings. Genotype abbreviations: 352 = 494352; 358 = 494358; BAL = Balami; BET = Beten; DAB = Dabbi; GEA = Gea-lamie; KARA = Karadebi; MAG = Magna; RED = Red dabi; ROS = Rosea; TUL = Tullu nasy; VAR = Variegata) and Group labels (CS = control shoot, SS = salt shoot). **a** Heat map showing accumulation of amino acids in tef varieties. The black box highlights the genotypes, Dessie in salt (DES-SS) and 494,352 in control (352-CS) showing a differential amino acid pattern. **b** Distribution of TCA, glycolysis, and sugar metabolites in tef shoot under control and 100 mM salinity

A PCA based on *m/z* features from the negative MS ionization mode of the shoot data showed a distinct difference between the control and salt-treated samples (Fig. 3a). The accessions, Dabbi, Gea-lamie, Red dabi and 494358 formed a distinct cluster from the remaining genotypes (Tullu nasy, Balami, Karadebi, Rosea, Variegata, Dessie, Magna, Beten and 494352) in both, control and salt stress. A total of 2049 m*/z* features were identified showing a significant change following salt treatment within the shoot metabolome**.** The pathway enrichment analysis indicated that the major sources of variation following salt treatment were linked to galactose metabolism, starch and sucrose metabolism, alanine, aspartate and glutamate metabolism, valine, leucine and isoleucine degradation and biosynthesis (Fig. 3b). A total of 156 m*/z* features from the identified pathways were annotated and displayed using a heatmap (Fig. 4a). The Red dabi and 494358 genotypes exhibited the most pronounced accumulation of all amino acids under salt stress. However, Dessie exhibited only a relatively minor increase in amino acids from a very low baseline so that the salt treated samples were grouped with the controls. A converse situation was seen with genotype 494352 plants germinated in water also showed high accumulation of phenylalanine, alanine, isoleucine, valine, and tyrosine and clustered with the stressed group (Fig. 4a). In addition to amino acid changes, metabolites associated with the tricarboxylic acid cycle (TCA cycle) and glycolysis significantly reduced in all genotypes with salt treatment. Other than Dabbi, Gea-lamie, Beten, Dessie and Variegata the levels of the TCA metabolite, citric acid, were up-regulated in all genotypes (Fig. 4b). Salinity reduced most of the sugars compared to control; however, important osmolytes including sucrose and trehalose, increased in the saline-treated group. Accumulation of the osmoprotectants sorbitol and trehalose differed between the varieties under salt stress. As compared to other varieties the shoots of Variegata, Beten, Magna and Rosea showed significant increases in sorbitol content with salt, whereas Dabbi, Gea-lamie, Tullu nasy and Karadebi showed higher trehalose contents.

### The root metabolome

**Fig. 5:**
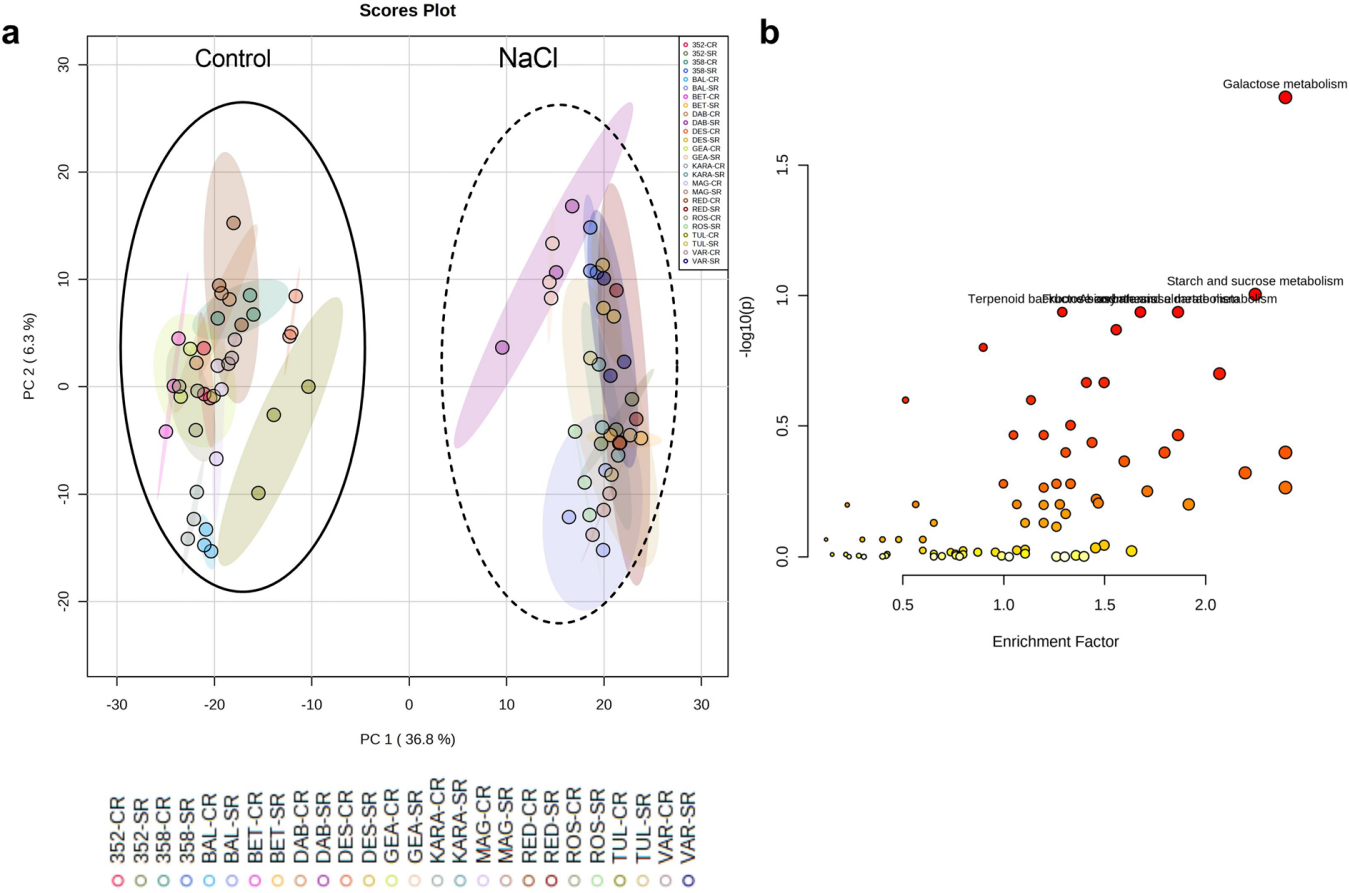
**a** Principal component analysis results for roots metabolome of thirteen tef genotypes. Genotypes: 352 = 494352; 358 = 494358; BAL = Balami; BET = Beten; DAB = Dabbi; GEA = Gea-lamie; KARA = Karadebi; MAG = Magna; RED = Red dabi; ROS = Rosea; TUL = Tullu nasy; VAR = Variegata; Groups: CR = control root, SR = Salt Root). The corresponding colour coded class legends are shown below the PCA. **b** Pathway enrichment of significant metabolite features in the root metabolome of tef seedlings

**Fig. 6:**
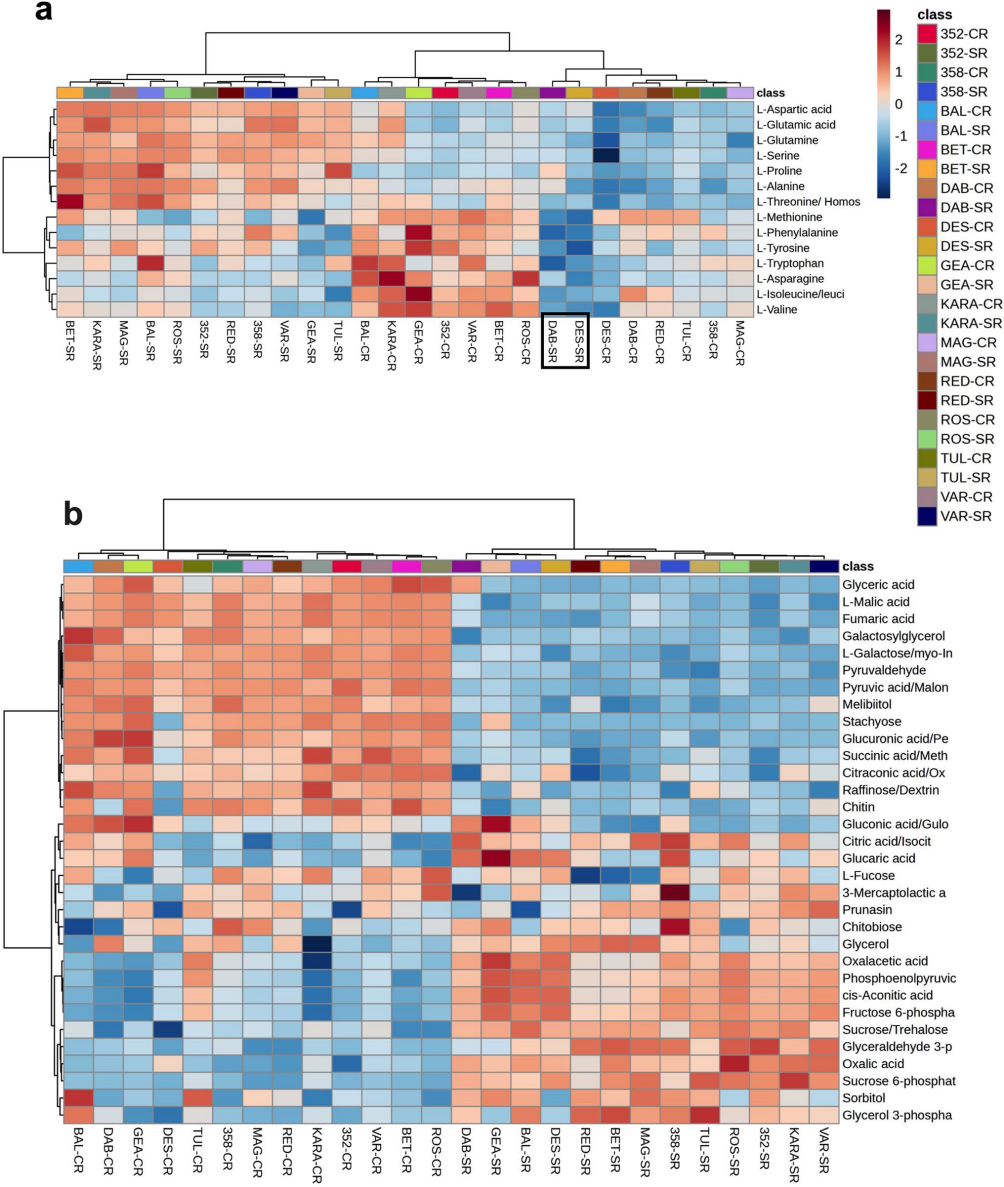
Heat map of the levels of significant annotated metabolites in the roots (R) of 13 tef varieties under control (C) and salt, 100 mM NaCl (S). **a** Levels of amino acids in roots. Genotypes: 352 = 494352; 358 = 494358; BAL = Balami; BET = Beten; DAB = Dabbi; GEA = Gea-lamie; KARA = Karadebi; MAG = Magna; RED = Red dabi; ROS = Rosea; TUL = Tullu nasy; VAR = Variegata; Groups: CR = control root, SR = Salt Root). The black box highlights DAB and DES with low level of amino acids under salt grouped along with control samples. **b** Heat map showing TCA, sugars, and glycolysis pathway related metabolites. Red colour in heat map indicates increased accumulation and blue indicates less accumulation of metabolites

PCA analysis of the root metabolome showed a clear separation between the control and salinity groups (Fig. 5a). A total of 1985 m*/z* features were found to be significantly different between control and salt treatments. Pathway enrichment analyses based on the metabolome of *Oryza sativa* library using the *mummichog* algorithm indicated the prominence of galactose metabolism, starch, and sucrose metabolism, terpenoid metabolism (Fig. 5b). Interrogation of these pathways identified 158 metabolites and the annotated compounds indicated that some of the key salinity induced metabolite changes in roots included increased accumulation of glutathione, oxidized glutathione, naringenin, proline, serine, glutamine, glutamic acid, aspartic acid, sucrose, oxalic acid, O-phospho-homoserine and sulfate. The two exceptions were the tolerant genotypes Dabbi and Dessie, where the accumulation of amino acids was lower in root compared to other varieties (Fig. 6a). However, malic acid, fumaric acid, glyoxylic acid, succinic acid, pyruvic acid, propionic acid, shikimic acid, 12-OPDA, ribose, raffinose, stachyose, glyceric acid, and shikimic acid were lower in roots of tef seedlings with salt (Fig. 6b, Fig. S1). We observed significant correlations between glutathione and phenylpropanoid derivatives which imply the role of secondary metabolites in conferring salt tolerance.

**Fig. 7:**
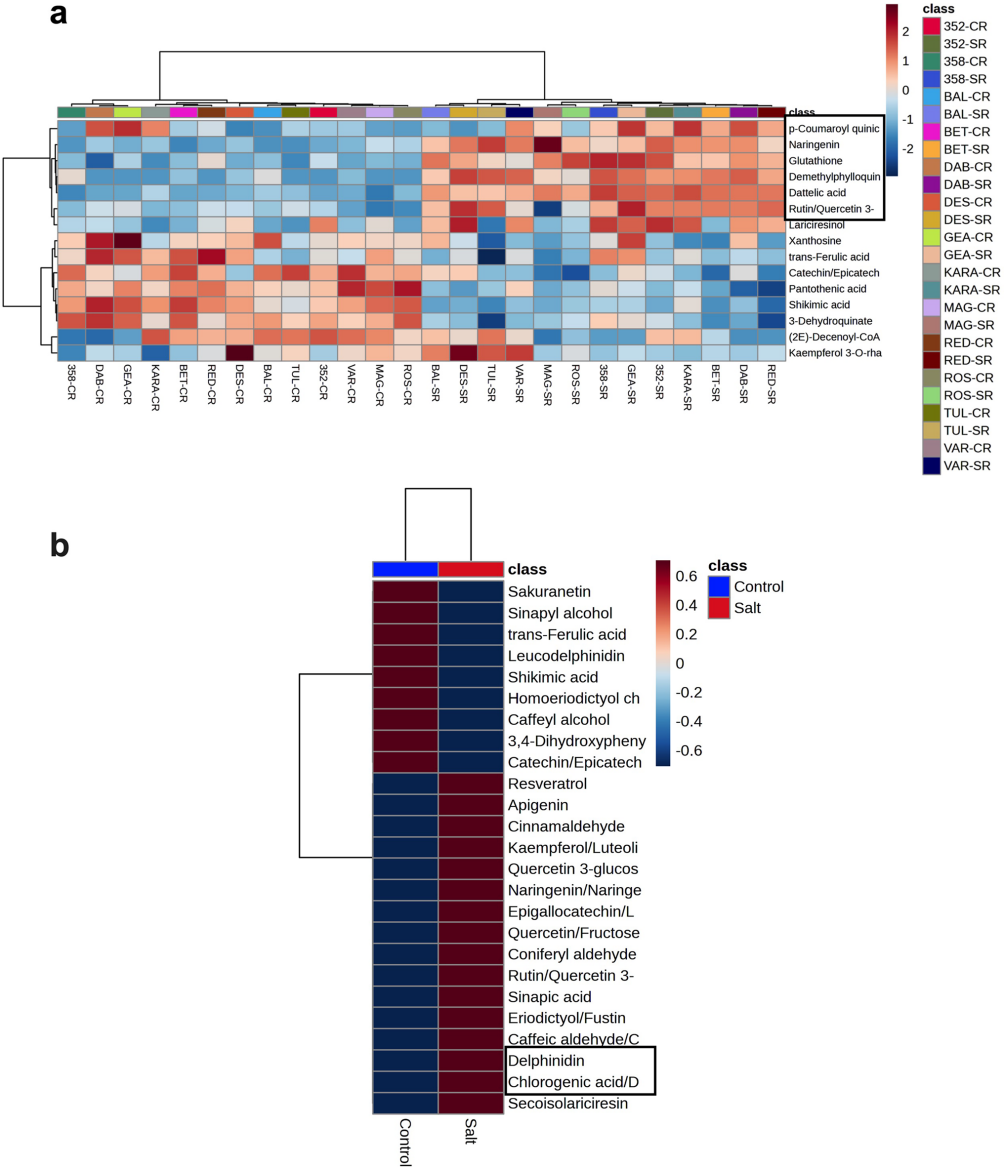
Differential accumulation of flavonoids. **a** Heat map of the level of flavonoid class of metabolites in tef roots under control and salt conditions. Black box indicates positive association of glutathione with flavonoid derivatives. **b** Pair wise comparison of flavonoid metabolites in control and salt treated roots of tef seedlings. The highlighted black box shows the level of delphinidin and chlorogenic acid

One of the interesting phenotypic observations was the presence of purple-red pigment in the roots under saline conditions. This colour could be associated with flavonoids and changes in flavonoid levels could be important in countering the oxidative effects of salt elicited reactive oxygen species (ROS) generation. To investigate this, we identified metabolites from the flavonoids class in the MS data matrix and their relative accumulation patterns were compared in salt treated and control roots (Fig. 7a). A pair-wise analysis showed significantly increased accumulation of phenylpropanoid and flavonoid derivatives such as resveratrol, apigenin, kaempferol, naringenin, rutin, quercetin 3-glucoside, epigallocatechin, sinapic acid, chlorogenic acid (CA) in the roots germinated under salt. We also detected high levels of delphinidin, a purple-coloured plant pigment in the salt treated root which could indicate the presence of pigmented root phenotype (Fig. 7b).

### Metabolite classes that may alleviate the negative effect of salinity in tef

**Fig. 8:**
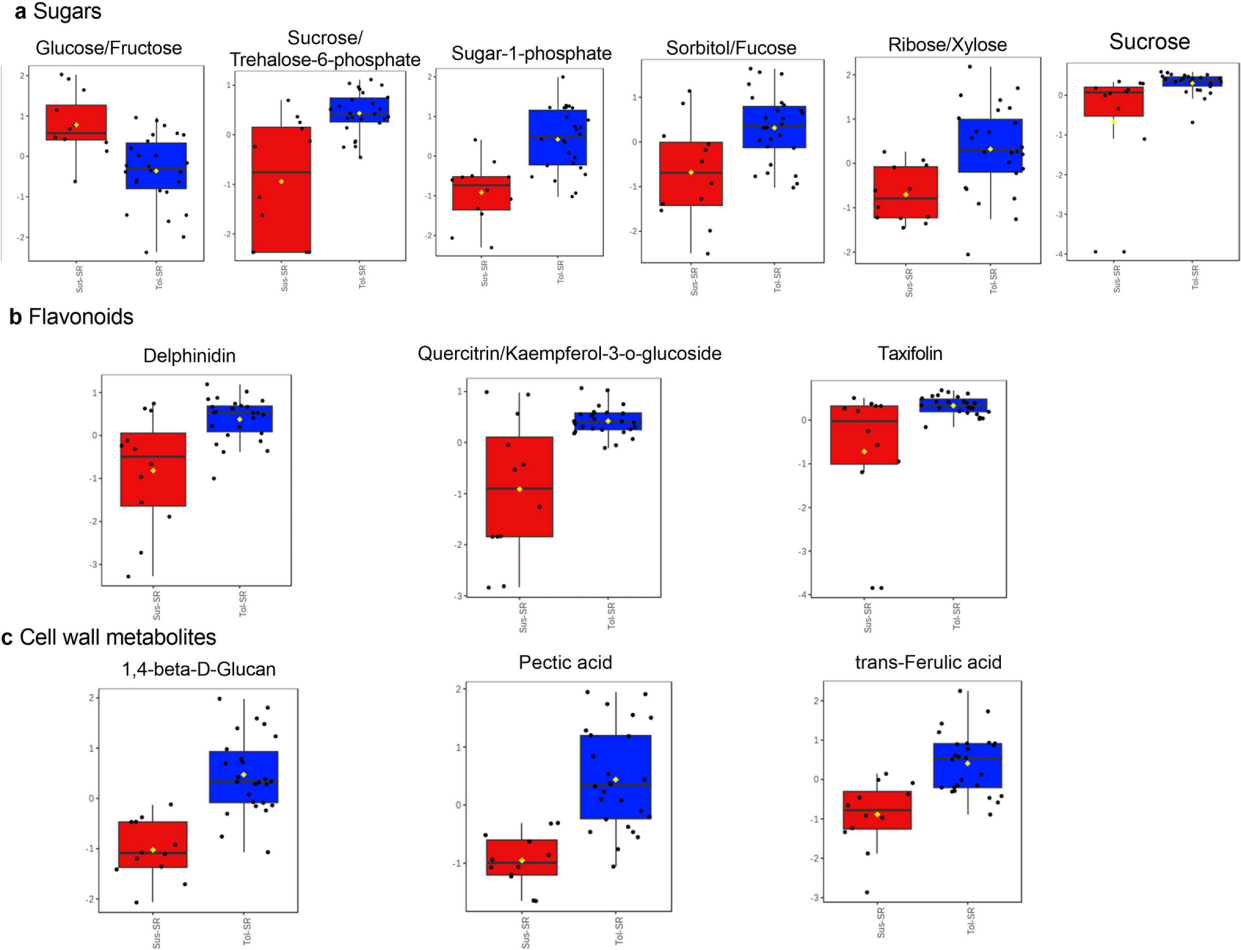
Pair wise analysis of root metabolites was done between the tolerant (Tol) and susceptible (Sus) under salt conditions (SR). Box plots showing levels of sugars (**a**), flavonoids (**b**), and (**c**) cell wall related metabolites in roots between tolerant (blue) and susceptible (red) tef genotypes

We next examined on the differential metabolites that could contribute towards relative salt tolerance of tef varieties which could suggest salt adaptive mechanisms in tef. We first examined the levels of proline, as a key stress-indicator. In both, shoots and roots of tef seedlings exposed to stress, the levels of proline were significantly elevated, although there was variety-specific variation (Fig. S2). We then undertook a pair wise comparison in an attempt to identify the metabolites that differed between tolerant and susceptible varieties. No significant differences were observed between the shoot metabolomes of tolerant or susceptible varieties but changes in the root metabolome could be linked to relative genotypic tolerance to salt (Fig. 8, S3a). The major sources of variation were identified based on t-test and included the lower levels of L-proline in the more tolerant varieties (Fig. S3b). Additionally, the more tolerant genotypes tended to show increases in sugars to counter the osmotic impact of salinity (Fig. 8a). Other likely important changes again included increase in flavonoids; delphinidin, quercitrin/kaempferol-3-O-glucoside and flavanone (Fig. 8b) that could aid in countering oxidative changes. Interestingly, there were also metabolites that could be linked to the cell wall associated changes (Fig. 8c) including pectic acid and ferulic acid which could modify the cell wall (Oliveira et al. 2020).

In order to explore the link between total flavonoid content and antioxidant activity, we performed DPPH and total flavonoid content (TFC) analysis in the shoots and roots of five tef varieties (Balami, Dabbi, Karadebi, Rosea and Tullu nasy). We found that the antioxidant activity was positively correlated with total flavonoid content within the tissue (R^2^ > 0.7). However, no significant differences were observed between the varieties under salinity (Fig. S3c).

## Discussion

Ethiopia ranks 7th in the world in terms of the total area affected by salt, especially the low lands and Rift valley (Adhanom 2019). Tef is a staple crop of Ethiopia and due to the increasing demand of tef as a healthy cereal, its cultivation is increasing year by year but compared to area of tef cultivation the yield is limited (Girija et al. 2022b; Woldeyohannes et al. 2022). To mitigate this, one possible solution is to expand the area of tef cultivation including the saline regions in Ethiopia. However, this can only be achieved with salt tolerant tef genotypes. At present there is no knowledge on the salinity responses of tef and such information would be especially important at the germination stage which is the most sensitive developmental stage to salinity (Bewley and Black 2013). Under saline conditions, seed germination is affected by ion toxicity induced by high concentrations of Na^+^ and Cl^−^ resulting in metabolic and ionic imbalance, lowering of osmotic potential, and ultimately oxidative stress (Tarchoun et al. 2022). Given this, there is a need to understand the salinity induced stress responses in tef. Here, we screened 19 tef genotypes for salinity tolerance to reveal the metabolite changes during germination and to predict some possible salt tolerant genotypes.

### Germination and growth response of 13 tef genotypes under salinity

Seed germination is the most vulnerable stage and exposure to environmental stresses at this stage will have a direct effect on crop development and productivity (Sudhakar et al. 2015). The germination rate and percentage vary between species, cultivars and also with increasing levels of salinity levels and osmotic stress (Kaveh et al. 2011). High levels of salt affect germination which was previously observed in wheat, rice, maize and Brassica species (Parihar et al. 2015). In our current study we also observed the same trend, where germination percentage varied between the 19 genotypes germinated at different NaCl levels. Germination was wholly inhibited at ≥ 250 mM NaCl in all genotypes. Based on the germination score these genotypes were divided into three groups, tolerant (Balami, Dabbi, Gea-lamie, Karadebi, Rosea, Variegata, Dessie, 494,352 and 494,358), susceptible (Beten, Tullu nasy, Red dabi, Magna) and very susceptible groups (Addissie, Alba, Enatite, Gommadie, Manyi, 3910). Previous germination studies using fifteen lowland tef genotypes at 2dS/m to 16dS/m salinity levels also showed that germination rate was affected by increased salt concentration (Asfaw and Itanna 2009). Having defined the salinity levels, we omitted the six non-germinated tef genotypes for further screening. Further screening showed varying growth response within the 13 genotypes at 100 mM NaCl. Most of the genotypes showed a germination score of 80 to 98% germination in 100 mM NaCl except for Beten, Tullu nasy, Red dabi and Magna (Fig. 1). One of the initial morphological response of salt stress is reduced growth rate which is caused by osmotic or water deficit (Khodarahmpour et al. 2012). In our study, we found a reduction in root growth and another striking phenotypic variation that we observed was the accumulation of purple-red pigment in roots under salt-stressed conditions. In a germination study of foxtail millet under salinity, root of one of the cultivars Yugu2 showed pink-red colour and the transcriptomic and metabolomic analysis implied the role of phenylpropanoid, flavonoid and lignin biosynthesis in conferring salt tolerance (Pan et al. 2020). Similarly, in tef, we observed the accumulation of purple-red pigment in the roots of all genotypes under salt except in Tullu nasy, Beten and Balami where the root colour was white and purple red in both control and salinity groups (Fig. 2). Root architecture and plasticity is very much regulated by water and nutrient uptake and it is the point of primary contact during seed germination (Arif et al. 2019). From the results, we speculate that the root color changes observed in tef are likely to be related to the response towards salt, but this needs to be further investigated.

### Flavonoid, osmoregulatory metabolites and cell wall changes in roots may counter salt -elicited oxidative events in tef

Flavonoids are a group of phenylpropanoid derived secondary metabolites including anthocyanins, flavonols, flavanols, condensed tannins and the level of these compounds varies between species, tissue, developmental stage and growth conditions (Kitamura 2006). Accumulation of flavonoids is a response to a wide range of abiotic stresses, such as drought, salinity, UV light (Saito et al. 2013; Khan et al. 2021). Previous studies investigating the effect of salinity on seed germination in millet, barley, and wheat have reported the up regulation of phenylpropanoid biosynthesis (Crizel et al. 2020; Hu et al. 2022). Further, stress can induce the expression of flavonoid biosynthetic genes like chalcone synthase (*CHS*)*,* a key enzyme in the biosynthesis of naringenin (Pi et al. 2016; Pan et al. 2020). Mechanistically, polyphenols and flavonoids can reduce the levels of salt-elicited ROS as shown, for example, during the seedling development of the extreme salt tolerant halophyte, *Salicornia brachiates* (Jacob et al. 2020).

Tef is a resilient crop and possesses high levels of endogenous flavonoids and phenols (Reta et al. 2022). In the current study, we observed increased in some phenolic compounds including quercetin 3-glucoside, rutin caffeic aldehyde, secoisolariciresinol, kaempferol/luteolin, chlorogenic acid, delphinidin, resveratrol, quercetin, apigenin, sinapic acid, naringenin, and eriodictyol/fustin in the roots of most of tef genotypes under salinity stress. In contrast, the levels of shikimic acid, ferulic acid catechin and leucodelphinidin were lowered (Fig. 7). Interestingly, the roots of tolerant lines exhibited prominent changes in certain flavonoids particularly delphinidin and kaempferol, hinting at a possible role in salt tolerance. Delphinidin is a blue to red colored pigment which is present in a variety of fruits and flowers. It is recognized as a natural pH indicator of acidic, basic, and neutral pHs, forming red, blue, and purple colours, respectively (Husain et al. 2022). However, kaempferol is a yellow colored flavanol but both compounds are flavonoids and are derivatives of naringenin.

Salinity disrupts the ion homeostasis leading to excess generation of ROS accumulation. Osmotic adjustment and antioxidant accumulation during stress is associated with the accumulation of osmolytes (Peña Calzada et al. 2022). Osmoprotectants are intracellular low molecular solutes and increased accumulation of these solutes is a common response to stress (Zulfiqar et al. 2019). Several amino acids have essential osmoprotectant properties in plants, for example, proline, glycine glutamine, alanine isoleucine, asparagine, glutamine, and serine (Chen et al. 2022). However, in the case of proline, this is also an indicator of relative stress in plants, as its lower levels in certain varieties could also be linked to tolerance (Verbruggen and Hermans 2008). Accumulation of amino acids in soya bean has been reported to act as buffering compounds in attenuating the effect of salinity (Abd El-Azeiz et al. 2021). Cysteine has a role in activating antioxidant mechanisms involving glutathione and, moreover, the cysteine also minimizes the impact of abiotic stress in crops (Hussein and Alshammari 2022). In the shoots of salt-stressed plants, the levels of glutathione were high as the product of cysteine metabolism (Bakhoum et al. 2019). In this study, the accumulation of amino acids in shoot and root tissues in face of salinity increased. However, there was also a genotype specific response which was related to Dessie. In Dessie germinated under control and salinity conditions, the accumulation of the amino acids was low such that the salt-stressed shoot was clustered with the control group. Low amino-N content in salt-stressed flax plants has suggested the down-regulation of protein synthesis (Hussein and Alshammari 2022). Also, Dessie is a developed USA variety for high forage quality and has adaptability to drought as well as wet soils, so we predict that differential responses of amino acids observed in Dessie could be genotype specific (Saylor et al. 2021).

Soluble sugars including sucrose, hexose, trehalose, raffinose, and sugar alcohols, such as sorbitol and glycerol, are also act as osmoprotectants and have important roles in the maintenance of cellular functions and photosynthetic proficiency (Nawaz et al. 2022). In salt-stressed shoots and roots we observed the accumulation of sucrose and trehalose which is likely to be a defense mechanism in tef to maintain the ionic balance. Trehalose application in crops like maize and rice was found to be beneficial as osmo-protectant and can activate anti-oxidant activities like superoxide dismutase, peroxidase and catalase (Yang et al. 2014). The increased levels of glutathione and oxidized glutathione in salt-stressed tef roots with increased levels of proline and trehalose strongly suggest changes in cellular redox homeostasis (Fig. 6) in response to NaCl treatment.

On comparing the root-specific metabolite changes between susceptible and tolerant genotypes we found some key changes that could be associated with their sensitivity to salt. Sugars (sucrose, ribose, sugar-phosphate), flavonoid derivatives (quercitrin, delphinidin, taxifolin) and sorbitol significantly accumulated in the roots of tolerant genotypes. The plant cell wall is made up of cellulose and pectin which act as a structural barrier to protect plants. The cell walls are subjected to modifications in the event of growth, development, abiotic and biotic stresses (Vaahtera et al. 2019). Salt stress affects the water equilibrium between the outside and inside of the cell, resulting in changes in turgor pressure and these can deform the cell wall affecting its interaction with the plasma membrane (Wang et al. 2016). This can have chemo-mechanistic effects to perturb pectin cross-linking, microtubule stability, and cellulose deposition patterns. In Arabidopsis, salinity triggers pectin methylesterase (PME) activation to induce salt-dependent responses (Gigli-Bisceglia et al. 2022). Similarly, we observed increased levels of pectic acid and glucan in the more tolerant tef genotypes indicating cell wall alteration to salt. Equally, elevated levels of ferulate (Fig S3) could alter the properties of the cell wall, for example increasing cross linking to affect mechano-sensing properties and salt tolerance (de Oliveira et al. 2015). Further investigation will be required to understand the cell wall specific changes in the root upon salt stress.

## Conclusion

To our knowledge, this is the first report in tef that establishes the salinity induced metabolite responses. Our initial study showed that severe salinity (≥ 250 mM) inhibits in vitro germination of tef seeds. Salinity induced responses included differential accumulation of osmolytes, and flavonoid derivatives in shoots and roots with increased accumulation of glutathione and proline which are indicators of oxidative stress. In comparing the root metabolome between tolerant and susceptible genotypes we identified significant changes associated with flavonoid derivatives along with cell wall related metabolites (pectin) that could play a role in reducing the ROS accumulation and could be important in conferring salt tolerance in tef. As an orphan crop, there is little understanding of the molecular responses of tef to salt. This current study provides insights into the biochemical pathways and predicted some potential tolerant genotypes that could be further validated by transcriptomics so that it can be used as candidate elite varieties in tef breeding program.

### Author contribution statement

AG, BSH and LM conceived and designed the research. RF conducted the germination experiment and metabolite extractions. BSH analyzed the metabolite data. DK, EW and BSH undertook DPPH, TFC assays and analyses. MB ran the samples by FIE-HRMS and processed the raw datasets. AG, BSH, RY, LM wrote and revised the manuscript. All authors read and approved the manuscript.

## Supplementary Information

Below is the link to the electronic supplementary material.

Supplementary file1 (DOCX 16 KB)

Supplementary file2 (XLSX 335 KB)

Supplementary file3 (PNG 646 KB) Heat maps showing the levels of top 60 significant annotated metabolites in roots of 13 tef genotypes in control and 100mM salinity treatment

Supplementary file4 (JPG 460 KB) Box plot showing variation of proline levels in shoots (**a**) and roots (**b**) of 13 tef genotypes in control and 100 mM salinity treatments. The box plot indicates log10 transformed values with FDR ≤ 0.05

Supplementary file5 (JPG 304 KB) **a** PCA showing the metabolite distribution in the roots of salt susceptible (Sus-SR) and tolerant (Tol-SR) tef varieties. **b** Box plot showing levels of proline in Sus and Tol roots. **c** Correlation analysis of total flavonoid content (Y-axis) and DPPH free radical activity (X-axis) in shoots and roots of 13 tef genotypes. The data shows positive correlation with R2 = 0.77

## Data Availability

All data generated or analysed during this study are included in this published article [and its supplementary information files].
