# Supplementary material for: Metabolomics targets tissue-specific responses in alleviating the negative effects of salinity in tef (Eragrostis tef) during germination
Source: Planta. 2023 Aug 19;258(3):67. doi: 10.1007/s00425-023-04224-x (PMC10439848; doi:10.1007/s00425-023-04224-x)
Supplement: Supplementary file 1 — Supplementary file1 (DOCX 16 KB) [file 425_2023_4224_MOESM1_ESM.docx]

Table S1 List of Tef accessions obtained from US-GRIN germplasm collection used for the salinity screening. The table shows the accession number, genotype name and seed colour.

| Accession number | Genotype Name | Seed colour |
| --- | --- | --- |
| 524434 | Addissie | White |
| 524435 | Alba | White |
| 524436 | Balami | Mixed (White-Brown) |
| 524437 | Beten | White |
| 524438 | Dabbi | Red |
| 524439 | Enatite | White |
| 524440 | Gea-lamie | Red |
| 524441 | Gommadie | Mixed (White-Brown) |
| 524442 | Karadebi | Red |
| 524443 | Manyi | White |
| 524444 | Rosea | White |
| 524445 | Tullu nasy | White |
| 524446 | Variegata | Brown |
| 3910 | 3910 | Brown |
| 557457 | Red dabi | Brown |
| 243908 | Magna | White |
| 618761 | Dessie | Brown |
| 494352 | 494352 | Brown |
| 494358 | 494358 | Brown |
