# Supplementary figures and images for: Metabolomics targets tissue-specific responses in alleviating the negative effects of salinity in tef (Eragrostis tef) during germination
Source: Planta. 2023 Aug 19;258(3):67. doi: 10.1007/s00425-023-04224-x (PMC10439848; doi:10.1007/s00425-023-04224-x)

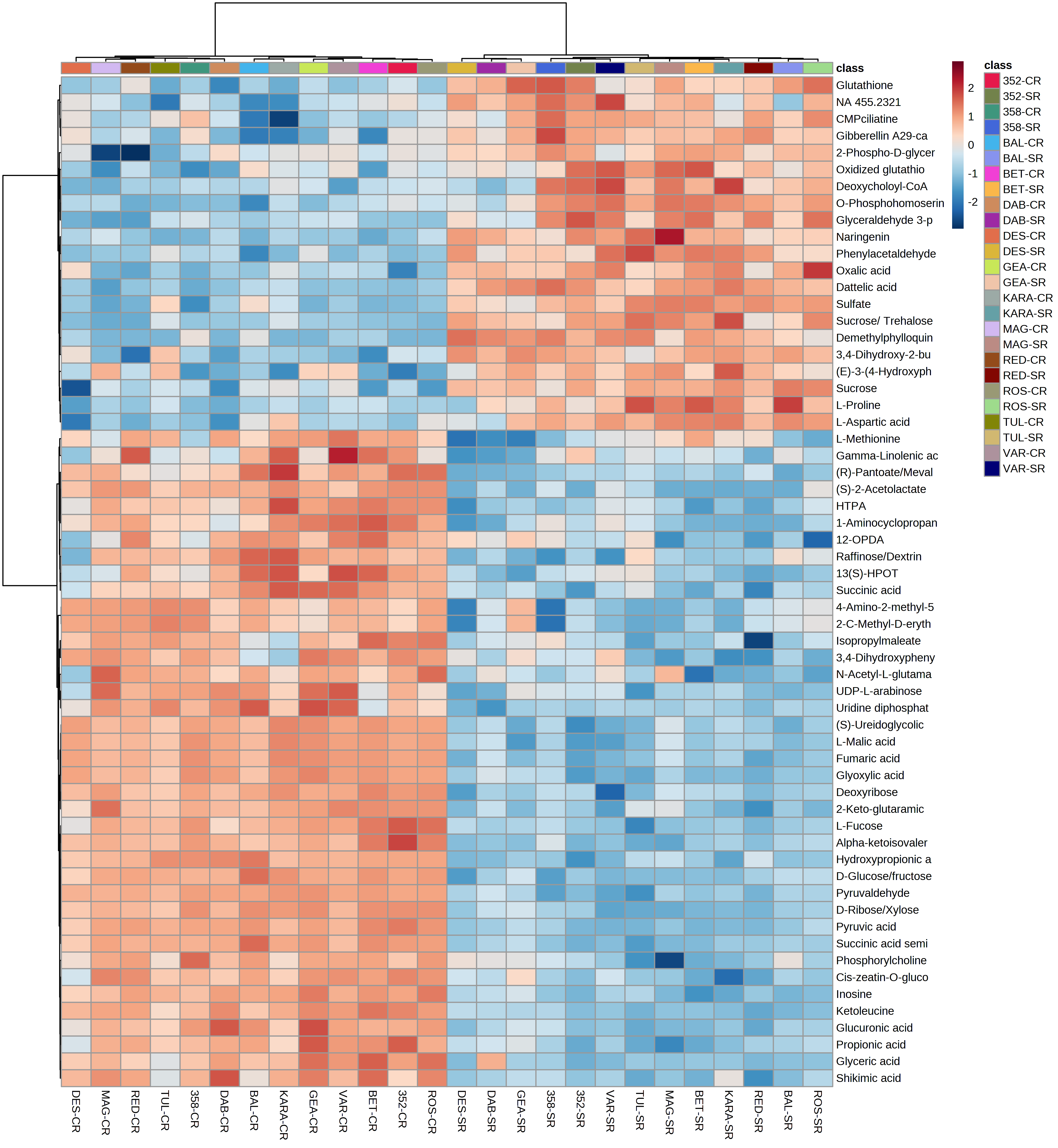

Supplement: Supplementary file 3 — Supplementary file3 (PNG 646 KB) Heat maps showing the levels of top 60 significant annotated metabolites in roots of 13 tef genotypes in control and 100mM salinity treatment [file 425_2023_4224_MOESM3_ESM.png]

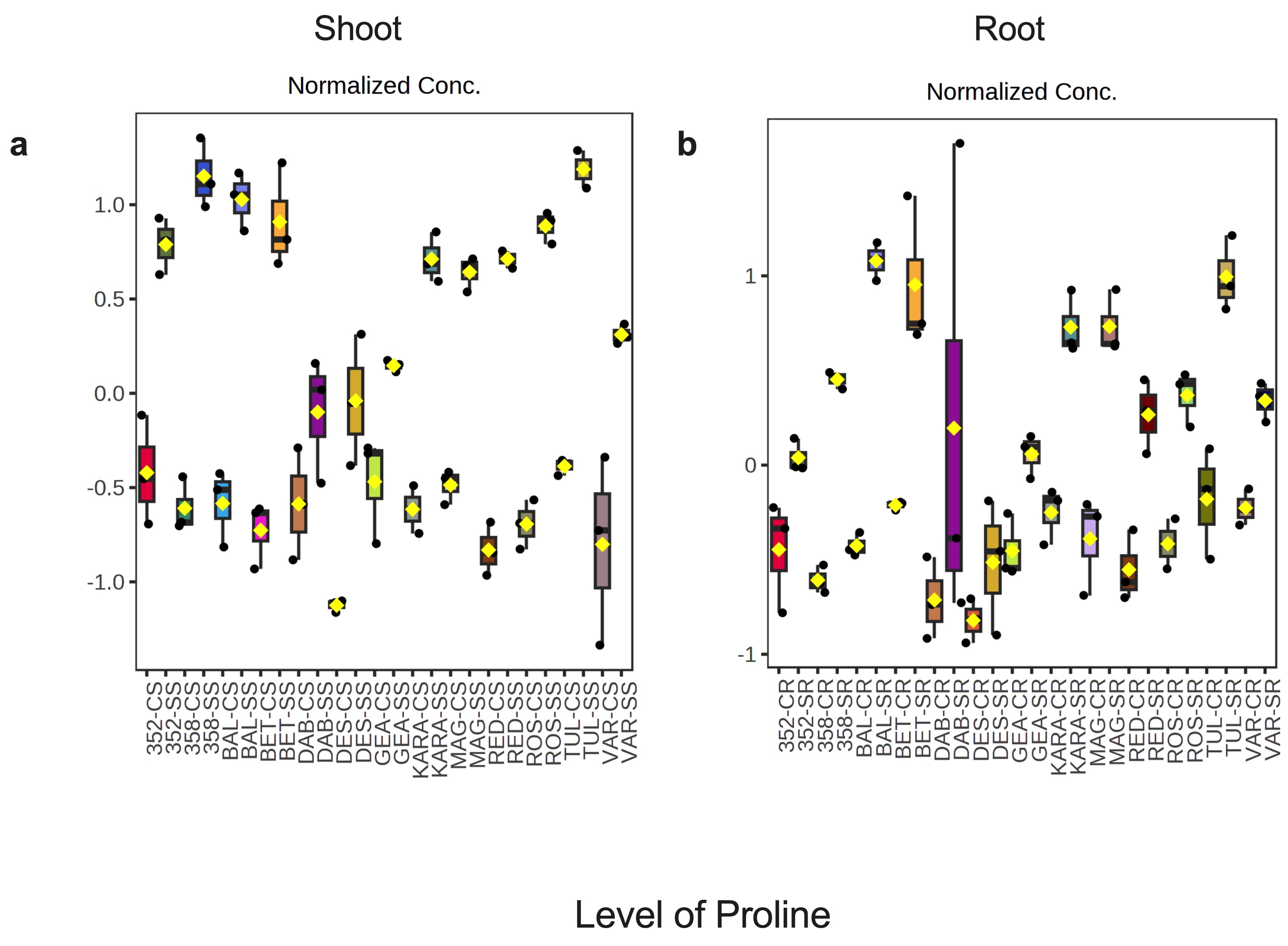

Supplement: Supplementary file 4 — Supplementary file4 (JPG 460 KB) Box plot showing variation of proline levels in shoots (a) and roots (b) of 13 tef genotypes in control and 100 mM salinity treatments. The box plot indicates log10 transformed values with FDR ≤ 0.05 [file 425_2023_4224_MOESM4_ESM.jpg]

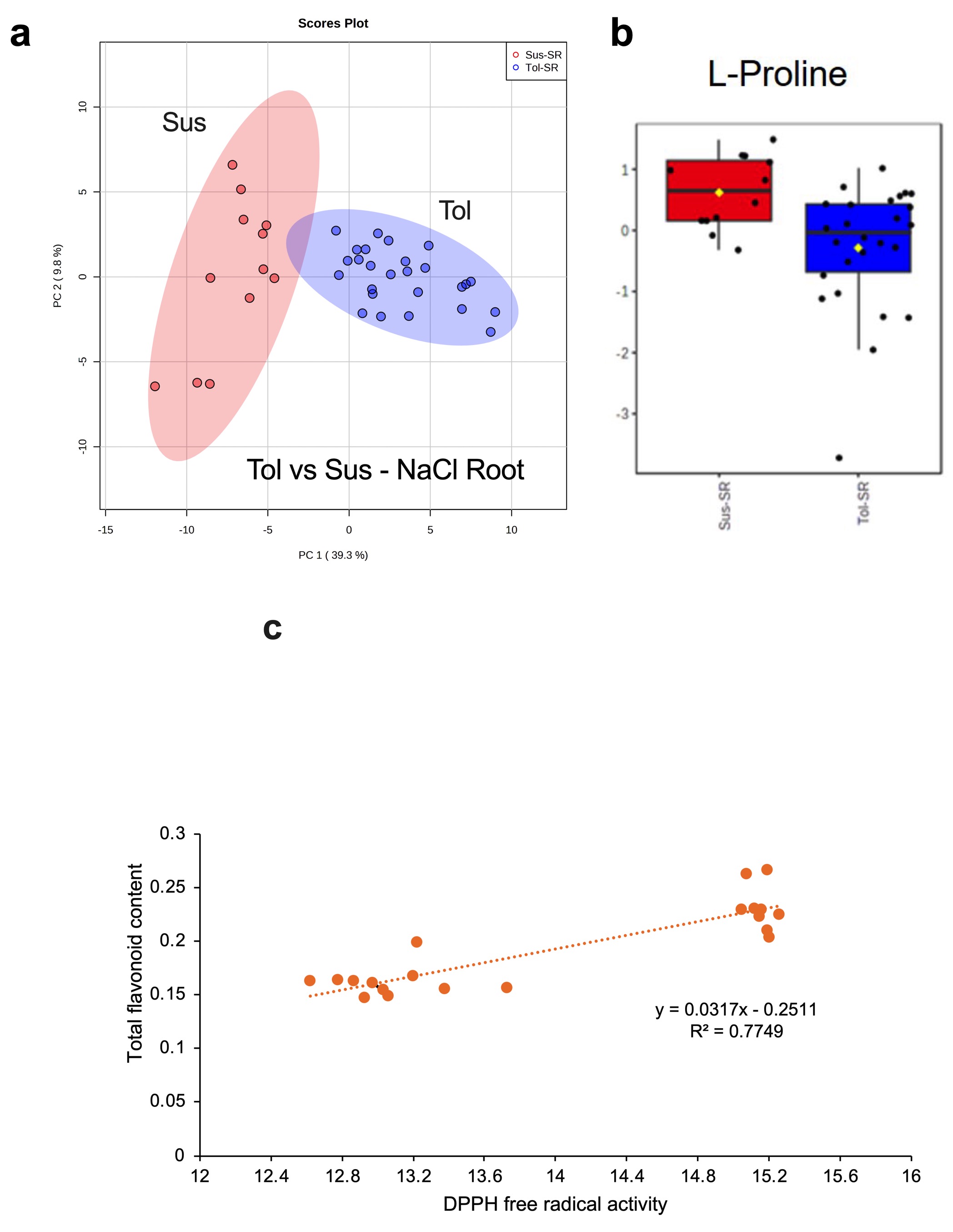

Supplement: Supplementary file 5 — Supplementary file5 (JPG 304 KB) a PCA showing the metabolite distribution in the roots of salt susceptible (Sus-SR) and tolerant (Tol-SR) tef varieties. b Box plot showing levels of proline in Sus and Tol roots. c Correlation analysis of total flavonoid content (Y-axis) and DPPH free radical activity (X-axis) in shoots and roots of 13 tef genotypes. The data shows positive correlation with R2 = 0.77 [file 425_2023_4224_MOESM5_ESM.jpg]
